# Prospects for Treatment of Lung Cancer Using Activated Lymphocytes Combined with Other Anti-Cancer Modalities

**DOI:** 10.3390/arm92060045

**Published:** 2024-12-06

**Authors:** Anastasia Ganina, Manarbek Askarov, Larissa Kozina, Madina Karimova, Yerzhan Shayakhmetov, Perizat Mukhamedzhanova, Aigul Brimova, Daulet Berikbol, Elmira Chuvakova, Lina Zaripova, Abay Baigenzhin

**Affiliations:** 1 JSC National Scientific Medical Center, Astana 010009, Kazakhstan; illak@mail.ru (M.A.); l.kozina@nnmc.kz (L.K.); madina.karimova.97@bk.ru (M.K.); e.chuvakova@nnmc.kz (E.C.); l.zaripova@nnmc.kz (L.Z.); a.baigenzhin@nnmc.kz (A.B.); 2 International Oncological Tomotherapy Center “YMIT”, Astana 010009, Kazakhstan; erzhan_1965@mail.ru (Y.S.); perizat07_04@mail.ru (P.M.); aigul_umit@mail.ru (A.B.); bericbol@mail.ru (D.B.)

**Keywords:** lymphocytes, lung cancer, activation, tumor, immunotherapy, immunosuppression

## Abstract

**Highlights:**

**What are the main findings?**

- The article explores the use of autologous activated lymphocytes with their ability to elicit robust anti-tumor immune responses as a promising complex treatment strategy for lung cancer.
- The application of various types of T-cell therapy in complex lung cancer treatment in >2000 patients, with a special focus on the autologous activated lymphocytes and infusion protocols, was examined and thoroughly discussed.
- The article emphasizes the benefits of combining activated lymphocyte therapy with existing treatments like radiation therapy, checkpoint inhibitors, and chemotherapy to improve efficacy and reduce resistance.

**What are the implications of the main finding?**

- Various cell types like natural killer cells, cytotoxic T lymphocytes, lymphokine-activated killer cells, and tumor-infiltrating lymphocytes contribute to immune responses that assist in eliminating cancer cells.
- The potential research gaps are identified, and a wider adoption of immune cell therapy as a component of combination strategies for the treatment of lung cancer is proposed.
- The article highlights ongoing research into the tumor microenvironment and its role in immune evasion, suggesting that targeting these mechanisms could boost treatment success, and discusses the significance of clinical trials in validating the effectiveness of activated lymphocyte therapies and the importance of real-world evidence in guiding clinical practice.

**Abstract:**

This review explores the significance and prospects of using diverse T-cell variants in the context of combined therapy for lung cancer treatment. Recently, there has been an increase in research focused on understanding the critical role of tumor-specific T lymphocytes and the potential benefits of autologous T-cell-based treatments for individuals with lung cancer. One promising approach involves intravenous administration of ex vivo-activated autologous lymphocytes to improve the immune status of patients with cancer. Investigations are also exploring the factors that influence the success of T-cell therapy and the methods used to stimulate them. Achieving a comprehensive understanding of the characteristics of activated lymphocytes and deciphering the mechanisms underlying their activation of innate anti-tumor immunity will pave the way for numerous clinical trials and the development of innovative strategies for cancer therapy like combined immunotherapy and radiation therapy.

## 1. Introduction

Lung cancer is one of the most widespread cancers. According to the Global Cancer Observatory (GLOBOCAN, data registries from 185 countries), in 2022, lung cancer accounted for about 2.5 million new cancer cases or 12.4% of all cancers, and 1.8 million deaths or 18.7% of all cancers, making it twice as deadly as the second deadliest cancer [1]. Lung cancer was surpassed by female breast cancer in 2020 [2] but returned to the leading position in 2022 [1]. According to the US cancer registers analyzed between 2018 and 2021 for both sexes, it took the second place with ~12% as most diagnosed and the first place with >20% in estimated cancer-related deaths [3,4].

The two main subtypes of lung cancer are small cell lung cancer (SCLC) and non-small cell lung cancer (NSCLC). NSCLC is the most common, comprising approximately 85% of all lung cancer cases [5,6]. All NSCLCs can be categorized into three main types: adenocarcinoma, large-cell carcinoma, and squamous-cell carcinoma. In the last decade, adenocarcinoma has replaced squamous-cell carcinoma as the most widespread (~40% vs. 25% of all NSCLCs) [7,8]. This is due to the opposite long-term trends in the prevalence of these subtypes, as shown, for example, in a large population-based study in the US [9]. Other studies reported even lower prevalence rates for squamous-cell carcinoma, e.g., 18% among all NSCLCs [6]. Identifying the subtype of lung cancer is important for designing effective treatment plans and prolonging patient survival. Currently, finding the right strategy for treating lung cancer remains a significant challenge that requires ongoing efforts in prevention, early detection, and development of innovative therapies [10,11].

The majority of lung cancer cases (more than half in total) are diagnosed at advanced stages, i.e., with distant metastases, which represents a significant challenge in the effectiveness of lung cancer treatment and leaves many patients with limited treatment options [12,13,14]. Only about a third of patients are diagnosed with stage I or II lung cancer, making them eligible both for biopsy examination and potentially curative procedures like lobectomy or mini-invasive video-assisted thoracic surgery [15,16,17]. Despite the relative stability of the combined diagnosed cases for NSCLC stages I and II at the level of ~35%, the proportion of stage I cases has remarkably increased in the last decade worldwide [16]. Based on available data in 2016 in the U.S., the most frequent single initial treatments for stage I were surgical resection (~60%) and radiation therapy (~25%), while for stage II, the surgery comprised ~25% of cases and only <15% of cases were radiation-based therapies; far fewer were for chemotherapy as a single treatment [16]. However, if all stages are analyzed, radiation therapy is almost twice as frequent (32%) than surgical resection (18%) [18].

For both early- and late-staged cancer patients, chemotherapy is another valuable option, especially in the progression of personalized medicine where a patient is prescribed personally effective medications [19]. As a single treatment, it was used at the rate of 15–25% for NSCLC stages IIIB and IV in the U.S. between 2010 and 2017; however, in combination with radiation therapy, the rate was much higher—e.g., for stage IIIA/IIIB it amounted to ~50% [16]. Similar to the U.S. data, the rate of people who have received at least one course of chemotherapy was about 55% in 2014 [20]. According to several clinical trials run in 2000–2010, stages I, II, and IIIA were considered eligible for adjuvant chemotherapy in resected lung cancer [15]. For patients with completely resected NSCLC, adjuvant chemotherapy has been established as a standard therapy that increases the 5-year survival rates by up to 15%, based on available data from several randomized trials [21]. A recent early-phase clinical trial showed that combined Nivolumab- and platinum-based neoadjuvant chemotherapy in lung cancer patients staged as IB and IIIA significantly prolongs event-free and overall survival rates [22].

On the other hand, single therapies have limited success rates and/or are accompanied by various adverse effects. Even after complete surgical resection, a substantial proportion of patients experience recurrence, often in distant organs, highlighting the aggressive nature of lung cancer and its ability to evade treatment [23,24]. Similar to resection, stereotactic body radiation therapy does not warrant recurrence even in NSCLC stage I/II in inoperable patients, with substantial cumulative rates of distant metastases reported by several clinical studies [25,26,27]. Moreover, to be effective for various subtypes of NSCLC, like squamous-cell carcinomas, radiation therapy should use higher doses of radiation [28,29]. Finally, non-combined chemotherapies are prone to cytotoxicity—the adverse effect that is almost always observed but may be mild or severe in different patients—or, as a more complicated and less predictable outcome, by multiple adverse effects related to the altered immune system responses due to the immunosuppression or immunostimulation [30,31]. For example, an analysis of 569 patients with advanced NSCLC or limited disease SCLC, performed for two randomized controlled studies, showed that 76.1% of them experienced severe toxic effects of chemotherapy, with 57.5% in the first course [32].

Immunotherapy represents a powerful alternative or adjuvant to radiation therapy and surgical resection, in particular, if non-resectable cancer is diagnosed. Immune surveillance is the main way to achieve the early detection and elimination of pre-cancerous cells in a healthy organism [33]. Normally, the immunosurveillance mechanisms and pathways work in a fine-tuned orchestration to prevent the growth and proliferation of pre-cancerous cells, mostly by sensing various factors released by these cells. However, if the immunoresistance of tumor cells outweighs immune cell activity, this fault gives a chance for cancer to develop [34]. The biological characteristics and diversity of the tumor cells play a pivotal role in its progression and resistance to specific treatments and therapies. The presence of heterogeneous cell populations within primary lung tumors, known as intratumor heterogeneity, poses a significant challenge for anti-cancer treatment [35,36,37]. In addition, the tumor cells directly (via gap junctions and connexins) and indirectly (via secreting various factors) interact with normal cells, thus significantly affecting the biomarker complexity of the tumor-localized immune reactions and promoting cancer spreading [38,39]. Therefore, the characterization of lymphocyte and macrophage populations in the tumor mass, as well as the spectrum of suppressive and regulatory molecules expressed by the intratumor immune cells, is a critically important stage before the application of individual immunotherapy [17]. Cancer cells, however, can evade immune surveillance and promote angiogenesis, leading to metastasis and recurrence [40]. It is also noteworthy to mention that apparently successful anti-tumor treatment in primary lung cancer may trigger metastasis growth months later, which is thought to be linked with treatment-induced damage of primary tumor tissue [41].

## 2. Immunotherapy Combined with Other Anti-Cancer Therapies

Addressing this complex problem requires a multi-faceted approach, including early detection through screening, personalized treatments tailored to the molecular characteristics of each tumor, and ongoing research to develop novel treatment strategies. This knowledge may facilitate further development of combination therapy, which may include immunotherapy, to effectively treat this complex disease and improve the quality and duration of life for people with lung cancer [42]. Although single immunotherapy has the potential to significantly improve survival for patients with NSCLC, patients often do not respond well to this therapy at the beginning, and resistance develops early in treatment or over time [43].

Inhibition of immune checkpoints, such as programmed cell death protein 1 and programmed death-ligand 1, have shown remarkable results in treating solid tumors [40,44] and recently became a standard first-line treatment for both NSCLC and SCLC [45], but its benefits are limited to a small number of patients [46,47] and there is also potential triggering of adverse effects in lungs under induced alterations in immune cells activity in the tumor microenvironment [31]. Also, in the patients who had advanced inoperable stage of NSCLC, were positive for programmed death-ligand 1, and received combined chemoradiotherapy, the overall 10-year survival rate was still very low [48]. Of note, the level of expression of programmed death-ligand 1 was found to be a significant risk factor for post-operative (after surgical resection) recurrence in NSCLC patients, therefore supporting the clinical value of personalized screening for specific molecular markers in order to select proper chemotherapy [49]. Another widely studied immune checkpoint, cytotoxic T-lymphocyte-associated protein 4 (CTLA-4), is expressed in regulatory T-cells (Tregs) at normal levels but becomes upregulated after T-cell activation. The suppression of the activity of the cells by inhibition of CTLA-4 has been shown to have prominent anti-tumor and anti-autoimmune effects [50], but it also resulted in high toxicity to the non-cancerous cells [51]. Recently, it was proposed to preserve rather than inhibit CTLA-4 to warrant safer cancer immunotherapy [52].

To overcome the problem of non-superior effects of single immunotherapy, researchers are investigating combining it with other therapies [53]. This combination strategy aims to enhance the immune system’s ability to fight cancer, potentially resulting in more effective and durable treatment prospects [54]. For example, in the US, the first-line treatment for advanced NSCLC has shifted from monotherapy aiming at the inhibition of the abovementioned immune checkpoints to combined chemo- and immunotherapy [55]. In addition, there are many approaches for treating cancer with drug therapy adjuvant to radiation therapy or chemotherapy, including the use of durvalumab [56,57,58]. The results of the PACIFIC trial show significant and long-term benefits for patients with stage III NSCLC who received durvalumab after chemoradiation, with significant overall survival and prolonged progression-free survival [56,58]. These findings lay the groundwork for future studies aimed at identifying the most effective combinations of therapies [56,57].

The combined use of immunotherapy and radiotherapy also creates a more effective immune response to cancer. On the one hand, radiation causes tumor cells to die through apoptosis (programmed cell death) as well as normalizes tumor vascularization, improving the oxygen supply and facilitating immune cell access. On the other hand, the radiation-induced dying of tumor cells promotes the release of apoptotic bodies, danger signals, tumor-associated antigens, and inflammatory cytokines. These signals activate dendritic cells and other antigen-presenting cells, which migrate to lymph nodes and present tumor-associated antigens, stimulating the production of specific T-cells. Finally, these activated T-cells target both the primary tumor in the radiation field and distant tumors [59].

Stereotactic body radiation therapy, also known as stereotactical ablative radiotherapy, is becoming increasingly popular due to its ability to deliver highly focused high doses of radiation with fewer treatments [60]. This method has shown excellent local tumor control and manageable side effects, and high-dose radiation in this therapy stimulates the immune system more effectively than traditional radiation therapy [61]. For example, only the combined use of focal radiation therapy and CTLA-4 inhibition therapy did induce activation of systemic anti-tumor T-cells in metastatic NSCLC, whereas the latter therapy alone was largely ineffective [62]. Numerous studies have shown that low-dose radiation therapy, defined as less than 2 Gy per fraction, can alter the tumor’s microenvironment effectively [63,64,65]. This facilitates the transformation of the stroma from immunosuppressing to immunostimulating, enhancing the efficacy of immunotherapy [66]. Interestingly, high- and low-doses appear to complement each other when combined, suggesting potential synergistic benefits [67].

The next sections of the review provide more detailed and comprehensive views on the most important elements of immunotherapies based on the anti-tumor-activated T-cell approach.

## 3. The Vital Role of T-Cells in the Treatment of Lung Cancer

The immune system’s response to tumor cells is important, but it does not warrant an adequate and effective antitumor response. Today, understanding the very nature of the complex cascade of interactions between the immune system and the tumor itself is an important issue in clinical research in the field of oncology. Following the approval of Nivolumab (2018) and Pembrolizumab (2019) for the treatment of NSCLC patients, clinical results have been obtained indicating significant improvement in many patient conditions, although some patients did not respond positively to this treatment [46,68,69]. To improve existing immune cell therapies and develop new treatment methods for patients with oncological pathology, it is crucial to understand how immune responses occur and are formed. Once this is understood, we can apply comprehensive treatments to cancer patients [70].

Due to the ability of T lymphocytes to destroy cells based on specific antigens, the immune system has become considered an opportunity to use it in the fight against cancer. The acquired knowledge of the molecular and cellular processes of blood lymphocytes contributed to the emergence of new treatment directions in the field of oncology and the development of cellular vaccines based on the blocking of control points. However, the outcomes of immunotherapies in solid tumors are, in general, less prominent compared to those designed for liquid tumors, mainly due to the complex and multifaceted effects of the solid tumor microenvironment [71]. Therefore, there is a high need for effective and low-toxic methods for treating lung cancer, which requires the development of novel methods using cell therapy.

The role of CD8+ T-cells in the immune response is currently well understood, while the function of CD4+ helper T-cells in this context is less well known. However, more and more evidence suggests that CD4+ positive T-cells still play a key role in the treatment of lung cancer, while a higher number of tumor-infiltrating CD4+ T-cells correlates with improved patient survival [70]. At the same time, CD4+ positive cells are essential for the “presentation” of dendritic cells by transmitting specific signals to CD8 cells, which allows them to recognize tumor cells and attack them. It is this process that allows the cross-priming of positive CD8+ T-cells, thereby increasing the ability to recognize tumor cells, attack them, and, most importantly, develop long-term memory of positive CD8+ T-cells [72]. Recent studies have shown that these positive cells also play an important role in recognizing mutated tumor antigens and leading to mutations in NSCLC tumors. This confirms that positive CD4+ T lymphocytes play an important role in activating the immune response to tumor cells and can be considered a new direction for the development and use of immunotherapeutic cell products in the treatment of tumors [73].

Previous studies have been based on the ability of positive CD8+ T-cells to recognize tumor cells through major histocompatibility complex (MHC) class I receptors and directly destroy them. However, as a result of numerous studies in this field, it has become known that the effectiveness of positive CD8+ T lymphocytes largely depends on CD4+ T-cells. These interactions involve antigen-presenting cells and are heavily dependent on interleukin-2 (IL-2) signaling.

Positive CD4+ T lymphocytes play an important role in increasing the activity of effector CD8+ T-cells in the following ways: (a) enhancing their propensity for killing tumor cells; (b) stimulating their proliferation, growth, and reproduction; and (c) attracting them to the location of tumor tissue [74,75]. This demonstrates the importance of positive CD4 + T lymphocytes in the blood in maximizing the effectiveness of CD8+ T-cell-based therapy and highlights the significance of interaction between these two types of T-cells for developing the most optimal treatment strategies [76].

Currently, adoptive T-cell therapy is used to amplify the natural activity of T lymphocytes. It consists of isolation of natural tumor-reactive T-cells from existing tumor tissues or blood (as a first step), genetic modification of these T-cells to give them specific recognition of tumor cells (second step), and transferring them back into the patient to augment the tumor-localized immune response [77]. While the adaptive transfer of tumor-infiltrating lymphocytes (TILs) has shown promising preclinical results, clinical trials have yielded uneven results [78,79]. Also, preparing a patient for chemotherapy before starting TIL therapy is recommended, which can significantly increase the efficacy of the treatment and lead to improved patient outcomes [80].

Since T lymphocytes play a crucial role in the development and progression of tumors, this makes them the most important targets for the treatment of oncological processes. T-cell-based immunotherapy is a promising way to develop additional approaches to the traditional treatment of lung cancer. Further research is needed to assess the full potential of immune cell therapy methods and improve clinical outcomes for cancer treatment [81].

Positive CD4+ T lymphocytes are important components of the adaptive immune system, working alongside positive CD8+ cytotoxic T lymphocytes. The ability of CD4+ T lymphocytes to differentiate into various specialized subtypes, each of which plays a crucial role in supporting other immune cells, leads to the strengthening of antitumor immunity in several ways. First, they enhance positive CD8+ cytotoxic T-cells and antibody response. Second, they secrete effector cytokines such as interferon-γ (IFN-γ) and tumor necrosis factor-α, which activate, direct, and regulate the immune response. Third, they provide direct cytotoxicity and destroy tumor cells, which makes them attractive targets for the development of immuno-therapy methods [81,82]. Attempts to use positive CD4+ T-cells against tumors included vaccination with epitopes with peptides designed to stimulate the production of specialized CD4+ T lymphocytes. These peptides were often derived from highly immunogenic tumor-associated antigens, such as representatives of the testicular cancer antigen family like NY-ESO1 [83].

Many studies have focused on measuring the increased production of tumor-specific cytokines by T-cells, in particular IFN-γ, as one of the markers of enhanced CD4+ antitumor reactions. Indeed, diminished expression of IFN-γ due to hypermethylation has been shown in cancer cells, which may contribute to the cancer progression and immune escape [84]; therefore, treatments resulting in up-regulation of IFN-γ by CD4+ T-cells, e.g., through demethylation or posttranslational histone modifications, seems to be an important component of the immunotherapy [85]. This approach is aimed at understanding and measuring the effectiveness of cellular vaccination in stimulating positive CD4+ T-cells to fight tumors [86].

## 4. Expanded Activated Autologous Lymphocyte Therapy

Cytotoxic T-cells and NK cells are the key effectors in the expanded activated autologous lymphocyte (EAAL) therapy used to stimulate the patient’s own immune components against cancer [30]. During 2 to 4 weeks of treatment in metastatic patients, this therapy resulted in the elevation of concentration of CD3+, CD8+, and CD56+ cells in the blood, as well as the number of IFN-producing cells. Importantly, this effect was observed in CD3+IFN+ lymphocytes and in CD3-IFN+ cells, i.e., EAAL therapy induced capacitation of T-cells and NK cells in producing IFN. Furthermore, there was a notable increase in the proportion of IFN-producing cells within the CD3+, CD8+, and CD3 cohorts following infusion. This suggests that EAAL treatment effectively boosted lymphocyte counts in peripheral blood, increasing the population capable of targeting and eliminating tumor cells [86].

The ability of NK cells to directly destroy target cells (NK-mediated cytotoxicity) can be potentiated via targeted expression of monoclonal antibodies on the surface of (pre)cancerous cells, i.e., through antibody-dependent cell-mediated cytotoxicity [87]. While studied in vitro, fresh peripheral blood mononuclear cells exhibit antibody-dependent cell-mediated activity, mediated by cetuximab, against a set of lung cancer cell lines [88] as well as against osteosarcoma [89]. These findings suggest that this treatment approach may strengthen the overall immune response, particularly with regard to NK cells. This allows for more efficient targeting and elimination of cancer cells.

A study conducted by Xie et al. (2019) investigated the safety and effectiveness of an immunotherapy approach using highly activated natural killer (NK) cells in 13 people with late-stage lung cancer [90]. The study involved harvesting NK cells from the patients’ blood, cultivating them for 12 days, and then reintroducing them into the patients’ bodies through intravenous infusion. To assess the impact of the treatment, the researchers evaluated various parameters both before and after the final infusion. This allowed them to monitor the patients’ response to the therapy and determine its effectiveness. In the study, various immune cell types were examined as well as levels of immune signaling proteins, carcinoembryonic antigen, and thymidine kinase 1. The CD3+CD56+ subset of cytokine-induced killer (CIK) cells, which are developed from blood and cultured with recombinant IL-2, was considered more effective against tumors due to its enhanced ability to target and destroy cancer cells. The presence of CD56 on these cells, which is a marker that may be related to cancer cell interactions, highlights the significance of these findings [90].

After a three-month follow-up, about 85% of patients maintained their stable condition, indicating that their cancer had not progressed. Conversely, about 15% of patients showed signs of disease progression. The level of IFN increased significantly after treatment, while the level of carcinoembryonic antigen decreased, which may indicate a reduction in cancer activity. Overall, the immune function of patients undergoing NK cell therapy remained stable [90]. The findings indicated that CIK cell therapy can enhance the effectiveness of conventional chemotherapy. Specifically, this treatment not only significantly improved immune function but also increased the total count of effector cells, which are responsible for directly attacking cancer cells, all without causing any major adverse effects [91,92].

A phase II clinical trial conducted by Li et al. showed the potential of CIK immunotherapy to augment the efficacy of standard chemotherapy in individuals diagnosed with NSCLC [93]. Their investigation has revealed important connections between CIK immunotherapy and several elements that might influence its effectiveness. Research by Chen et al. showed that the expression levels of MHC class I-related chain A in patients with gastric cancer correlated with their responses to CIK treatment. Patients with higher MHC class I-related chain A levels were more likely to experience positive outcomes from this therapy. A phase II/III study demonstrated that the combination of radiofrequency ablation and CIK therapy was both safe and effective for individuals suffering from colorectal liver metastases. Several clinical studies have explored the effectiveness of CIK and dendritic CIK cell therapies in lung cancer. Overall, the findings consistently support the effectiveness of these treatments for particular forms of cancer [93,94].

These outcomes highlight the potential of CIK immunotherapy as a promising alternative for treating cancer, particularly lung cancer. Further research is needed to thoroughly understand the mechanisms behind its effectiveness and evaluate its role in personalized medicine strategies [95,96]. In the study by Zhang et al. (2015), the successful use of EAAL for the treatment of SCLC was described [96]. This research comprised 32 patients diagnosed with SCLC and separated them into the EAAL-receiving group and the control group. The procedure for obtaining EAALs included several key steps. Blood was collected in volumes ranging from 20 mL to 100 mL with peripheral blood mononuclear cells (PBMC) isolated using the Ficoll-hypaque gravity centrifugation technique. The isolated PBMCs were cultured for 14 days in specific medium (IMSF-10) with cytokine IL-2, which facilitated the proliferation and activation of immune cells. This method resulted in an increased population of EAAL. Before administering the EAALs to patients, distinct types and properties of the cells were identified by thorough characterization [97]. Following the cultivation and in vitro expansion, a substantial increase (*p* < 0.01) was noted in the percentages of CD3+, CD3+CD8+, CD45RO+, CD28+, CD29+, CD8+CD28+, and CD3+CD16+/CD56+ cells. In contrast, there was a significant reduction in the percentages of CD19+, CD3+CD4+, CD45RA+, CD4+CD25+, CD4+CD29+, and CD3-CD16+/CD56+ (NK cells). After the in vitro culture and expansion, notable rises (*p* < 0.05) were observed in the percentages of CD3+ (T-cells), CD3+CD8+ (cytotoxic T-cells), CD45RO+ (memory T-cells), CD28+ (co-stimulatory receptor T-cells), CD29+ (integrin for cell adhesion), CD8+CD28+ (co-stimulatory receptor cytotoxic T-cells), and CD3+CD16+/CD56+ (T-cells showing NK cell markers). The Kaplan–Meier analysis of survival demonstrated that the median survival time as well as 1- and 3-year survival rates tended to be longer in EAAL-treated patients compared to controls, although the differences were not statistically significant. The results suggested that EAAL may contribute to enhanced survival in SCLC patients. Additional analysis using multivariate Cox regression revealed that the number of chemotherapy sessions and the use of EAAL immunotherapy are both independent factors predicting survival in patients with SCLC. This implies that both factors have a significant effect on survival, even when accounting for other variables. The study demonstrated the effectiveness and safety of in vitro expansion and induction of EAALs. These findings support that adoptive immunotherapy using EAALs can prolong overall survival in SCLC patients [96,97], but further investigations with increased sample size are necessary to verify these findings.

The principal challenges in the clinical use of antigen-specific T-cells include expanding these cells to obtain a sufficient quantity while minimizing the presence of Tregs, preventing a reduction in their function, and determining their role and position in combined tumor immunotherapy. Moreover, there is a need to establish the most efficient protocols for their deployment. To encourage the effective proliferation of antigen-specific T-cells, various cytokines and their combinations were used by different researchers.

It was demonstrated that in addition to the traditionally employed IL-2 for activating adoptively transferred T lymphocytes, IL-21 and homeostatic cytokines (like IL-7 and IL-15) should also be used because of their ability to encourage the maturation of memory T-cells [98]. However, the cytotoxic activity of antigen-specific T-cells (both cytotoxic T lymphocytes and helper T-cells) is not influenced by commonly used modulators of cytotoxic anti-tumor responses such as pro-inflammatory cytokines (IL-12 and IL-18), indoleamine 2,3-dioxygenase inhibitors (L-methyl-1-tryptophan), and cyclooxygenase-2 inhibitors (celecoxib) [99]. Nonetheless, the analysis of cytotoxic factors (Fas ligand, TNF-related apoptosis-inducing ligand, perforin, Granzyme B) and cytokine secretion showed a significant rise in the TNF-related apoptosis-inducing ligand-positive CD8+ lymphocytes and cells producing IFN-γ in NSCLC. Simultaneously, there was no stimulation of IL-4 and IL-10 production. These observations suggest a dominant activation of the type 1 T helper immune cellular response, implying that these modulators may be more effective in steering the immune response toward a profile with dominance of type 1 T helper, which is generally perceived as favorable for anti-tumor immunity [99].

The same study identified that epitope-specific lymphocytes isolated via magnetic separation and cultured with a cytokine mixture exhibited notably higher cytotoxic activity against tumor cells compared to activated mononuclear cell cultures and dendritic cells [99]. The research used mouse splenocytes, specifically CD8+ T-cells, which were programmed to recognize specific epitopes presented by MHC-I. The researchers successfully developed a rapid ex vivo expansion method followed by cryopreservation to obtain and store antigen-specific T-cells. This approach maintains cell viability and cytokine production, as well as the capacity of T-cells to differentiate, migrate, and infiltrate tumors, leading to tumor regression [100,101]. Cryopreservation does not notably impair the key properties of cytotoxic T lymphocytes, despite some studies suggesting otherwise [100]. These findings support the use of cryopreservation as a viable technique.

It is well known now that the activation of CD8+ T-cells by various signaling cascades and molecular factors within the tumor microenvironment plays a key role in the normally occurring inhibition of tumor immunity as the most powerful inherent anti-tumor effector [102,103]. In vitro “activation” of T-cells taken from the patient uses methods of genetic engineering to trigger the expression of specific proteins on the surface of these T-cells [104]; this groundbreaking methodology resulted in the rapid evolvement of so-called adoptive T-cell immunotherapy in the treatment of cancers [105]. The infused activated CD8+ T-cells can help enhance the suppressive and cytotoxic actions of the patient’s immune system through repeated infusions. This indicates that repeated infusions can build and strengthen the immune response. Of interest, different populations of CD8+ cells, if subtyped by the expression level of programmed cell death protein 1, provide different anti-cancer activity against NSCLC cells, as shown in cultured cells [106]. Additionally, the infused adoptive T-cells, including central memory CD8+ T-cells, can persist and accumulate in the body over time. This results in an increase in the total quantity of CD8+ T-cells and a reversal of the CD4:CD8 ratio in peripheral blood. This occurrence has also been observed in other clinical studies, indicating that adoptive T-cell therapy can have lasting effects on the immune system [107].

Research has shown that T-cells isolated from patients in the early stages of the disease often exhibit a stronger anti-tumor response compared to those isolated after successful treatment [107]. This suggests that the tumor microenvironment and the advancement of the disease can impact the effectiveness of T-cells. Possible explanations for this phenomenon include (a) exhaustion or energy when T-cells can become exhausted or unresponsive due to prolonged exposure to tumor antigens; and/or (b) the influence of the tumor microenvironment, which can suppress the function of T-cells, particularly through the presence of Tregs. These findings could explain the limited success of lymphokine-activated killer (LAK) and TIL therapy, as higher levels of Tregs have been observed during cultivation and in TILs. However, the role of Tregs in these therapies needs further exploration.

It has been found that during long-term cultivation ex vivo, LAK cells remain at the baseline level despite the increase in the amount of Tregs [108]. Others showed a decrease in the functional activity of T lymphocytes against the background of cultivation with IL-2, since IL-2 accelerates the aging process and the formation of the T-regulatory immunophenotype [109]. Some authors propose a method of immunomagnetic separation, which promotes the acceleration of tumor regression due to the elimination or selective inhibition of Tregs [110]. At the same time, in addition to the factor related to Tregs, other factors also take place when introducing them into the body, reducing the activity of lymphocytes activated ex vivo. One of them is CIK cells, obtained by culturing in vitro PBMCs with specific cytokines. As a result, a wide range of cells appears, most of which express CD3 and CD56 markers, the so-called natural killer T (NKT) cells. These NKT cells have a unique ability aimed at cell proliferation and cytolysis, thereby bypassing the MHC, and they can recognize and kill target cells [111].

The use of a combination of chemotherapy with adoptive immunotherapy with autologous CIK cells in lung cancer patients was compared to those who received chemotherapy only [112]. After 14 days of in vitro incubation, the CIK content was significantly higher in the study group by the percentage of CD3+, CD3+CD8+, CD3+, CD56+, and CD3-CD56+ cells, demonstrating the high potential of this immune cell population. Moreover, these data were correlated with clinical symptoms in the patients in the CIK treatment group toward improvement. No adverse events or serious toxicities were observed during the study when using autologous CIK cells in combination with chemotherapy. The group where patients received a combination of CIK and chemotherapy demonstrated significantly better survival rates compared with the control group. According to the study results, the median progression-free survival at 3 years and 5 years was 44.7% and 26.8%, respectively, in the CIK group compared with the control group and was significantly higher, and the median overall survival at 3 years and 5 years was 74% and 62%, respectively, in the CIK group, which was also higher than in the control group (*p* < 0.014). These results indicate that the use of autologous CIK cells in combination with chemotherapy can improve the response to treatment, progression-free survival, and overall survival in patients with lung cancer [112].

Cellular immunotherapy for CIK typically uses similar methods to extract these cells. Autologous CIK cells are obtained from peripheral blood by activating and expanding the patient’s own PBMCs ex vivo and then reintroducing them back into the patient. CIK cells, often referred to as NKT cells, can be greatly amplified, 200- to 1000-fold, within 14–21 days of culture. This growth is achieved by initially stimulating the cells with CD3 antibodies and various combinations of cytokines. Ex vivo expanded CIK cells are characterized by the expression of CD3 and CD56 markers. They exhibit potent cytotoxic activity against various tumor cell lines and animal models bearing the tumor antigen [113]. There are several clinical studies shown that combining CIK immunotherapy with chemotherapy offers potential benefits over chemotherapy alone in patients with advanced NSCLC [114]. Immunotherapy represents a promising avenue for cancer treatment by selectively targeting and killing cancer cells while leaving healthy cells and tissues unharmed. Recent advances in clinical trials have fueled enthusiasm for combining adoptive cellular immunotherapy with traditional treatments to achieve potent, effective, and long-lasting clinical responses [114].

In addition, many studies have described the effect of immune cell therapy on reducing the resistance of patients’ tumor cells to chemotherapy. The combination of chemo- and immunotherapy has the potential to mitigate the side effects of traditional chemotherapy, improve the patient’s quality of life, and prolong the survival of patients with advanced NSCLC. For example, a combination of norelbine-platinum chemotherapy followed by vaccination with autologous carcinoembryonic antigen peptide-treated dendritic cells and CIK cells produced relatively mild side effects and was well tolerated by most patients even after four cycles, each of 30 days [115]. Despite this, allergic reactions (rash, itching) and non-infectious fever were significantly more frequent in the chemoimmunotherapy group (64.2% and 71.4%, respectively) compared to the control chemotherapy-only group (7.1% and 21.4%). On the other hand, grade 3/4 fatigue was significantly less common in patients receiving chemoimmunotherapy compared to the chemotherapy group—7.1% and 57.1%, respectively [115].

Another study investigated the use of CIK cells in patients with advanced SCLC where the experimental group received chemotherapy plus CIK cell transfusion and the control group received chemotherapy alone [116]. The results showed a significantly higher overall response rate in the combination treatment group (40.9%) compared to the control group (9.1%). This indicates that the addition of CIK cell therapy to chemotherapy significantly improved the treatment response in patients with advanced SCLC. The study also showed that progression-free survival was significantly longer in the combination treatment group (8 months) compared to the control group (4 months). Importantly, no serious adverse events were observed after CIK cell infusion. These results strongly suggest that the combination of chemotherapy with CIK cell immunotherapy may be a safe and effective treatment option for patients with SCLC [116].

In a phase II clinical study, the use of autologous immunotherapy with CIK cells was found to enhance the effectiveness of standard chemotherapy in patients suffering from advanced NSCLC [114]. The stimulation of CIK cells by dendritic cells significantly increased the antitumor effects, and the integration of chemotherapy with CIK/dendritic cell treatment led to improved clinical results for individuals with advanced NSCLC [117,118]. CIK cells are produced from peripheral lymphocytes using a combination of cytokines, which includes CD3 monoclonal antibodies, IL-2, and IFN-γ [119]. Moreover, pairing CIK cells with endostatin or dendritic cell-based cancer vaccines might demonstrate a synergistic benefit in enhancing clinical outcomes. In recent years, CIK cells have been widely employed as an immunotherapeutic approach for various malignancies due to their strong expansion and cytotoxic functions, especially when activated by dendritic cells [120].

## 5. Adoptive Immunotherapy of Cancer Using Tumor-Infiltrating Lymphocytes

Adoptive immunotherapy using T lymphocytes that were cultured for extended periods of time and were dependent on IL-2 was introduced in the late 1980s. In one of the early studies, these T-cells were administered to individuals suffering from metastatic lung adenocarcinoma, having been extracted from cancerous tissue explants and cultivated in a medium enriched with recombinant IL-2 [121]. The T lymphocytes displayed activation markers and exhibited the capacity to target and eliminate a wide range of tumors. Upon intravenous injection, indium-tagged T-cell blasts mainly concentrated in the lungs, liver, and spleen. Although external imaging revealed a low count of infused lymphocytes at tumor locations, five out of seven patients noted a decrease in their cancer. However, none of the patients experienced a reduction exceeding 50% of their overall tumor volume. Furthermore, three patients showed increased delayed hypersensitivity reactions to protein antigens after therapy. This suggests that tumor-derived T-cells, when cultured for a long time, can be safely injected into human patients and potentially enhance immune responses, helping to reduce tumors in vivo [121].

The ex vivo expansion and reinfusion of TILs have been effectively implemented for treating various cancers, usually involving IL-2 for TIL expansion and subsequent support of these cells after reinfusion. Despite this, the use of IL-2 poses challenges due to its inclination to amplify Tregs and myeloid cells preferentially, along with its systemic side effects [122]. Furthermore, the efficacy and safety of lifileucel (LN-145), a cell therapy based on autologous TILs, were evaluated in patients with metastatic NSCLC who had shown progression after prior immunotherapy [78]. The cell product utilized in this research was derived from tumor tissues, mainly from lung specimens across various anatomical regions. The treatment showed efficacy against tumors that are often resistant to immunotherapy, including those lacking the expression of programmed death-ligand 1, with low mutational burdens, and harboring the mutation in gene-encoded Serine/threonine kinase 11. Adverse effects were mostly predictable, and two patients succumbed to treatment-related complications: cardiac failure and multi-organ failure. Another recent study reported a measurable objective response rate only in each fifth patient with advanced NSCLC treated with lifileucel [78]. Nevertheless, lifileucel exhibits promise as a viable therapy for patients with metastatic NSCLC who have not responded to prior treatments [78].

The presence of TILs has been found to correlate positively with lung cancer prognosis, suggesting that lung cancer is an ideal candidate for adoptive TIL therapy [123]. This approach entails the extraction and expansion of tumor-specific lymphocyte populations in vitro. Recent studies illustrate the potential of TIL-based therapies. For example, a 2021 phase 1 trial evaluated the safety and initial efficacy of TIL therapy combined with Nivolumab in late-stage NSCLC patients who had previously undergone Nivolumab monotherapy [79]. Patients received lymphodepletion therapy using cyclophosphamide and fludarabine, followed by TIL adoptive cell transfer alongside IL-2, and subsequently underwent maintenance therapy with Nivolumab. The results showed that in most participants, the combined therapy was generally safe and clinically active. TILs showed longer engraftment when compared to those infused into untreated patients, and the level of engraftment correlated with clinical outcomes. However, prior lymphodepleting chemotherapy to induce concurrent host immunosuppression played a vital role in TIL infusion with high-dose IL-2 immunotherapy and, therefore, needed to be implemented [79]. Also, in many cases, the anti-tumor effects were temporary, with a lack of sustained persistence of the transferred cells [124].

## 6. Lymphokine-Activated Killer Cells

Regarding lymphokine-activated killer (LAK) cells, which can non-specifically target both autologous and allogeneic tumor cells, early attempts showed promising results in treating advanced cancers. A study involving 121 patients with malignant effusions due to advanced lung cancer indicated that the combination of autologous LAK cells and recombinant IL-2 resolved effusions in 58.6% of cases and significantly reduced them in 36.2% [125]. Nevertheless, high IL-2 doses pose challenges due to serious side effects such as capillary leak syndrome, which can complicate LAK cell therapy in clinical practice. Another clinical trial involved 105 patients who underwent surgery for incurable primary lung cancer and were randomly assigned to two groups [126]. The group receiving a combination of recombinant IL-2 and LAK cells, in addition to radiation or chemotherapy, exhibited improved 7-year survival compared to the control group receiving radiation or chemotherapy alone [126]. Similar results were observed in a randomized phase III study of the treatment of primary lung carcinoma [127].

Despite these promising findings, the use of LAK cells in clinical settings faces a significant hurdle: the high doses of IL-2 often lead to serious side effects such as capillary leak syndrome [128]. This syndrome can cause hypotension, oliguria (reduced urine output), pulmonary edema, and dyspnea (difficulty breathing), making it a major obstacle in the development of LAK cells for clinical use. While LAK cells exhibit remarkable cytotoxicity against cancerous cells in vitro, clinical trials have yielded disappointing results, with only a handful of exceptions. The key point for their inability to exhibit cytolytic activity is their lack of specificity, which prevents LAK cells from selectively accumulating in tumor tissue.

Kimura et al. (1997) demonstrated the effectiveness of adjuvant LAK cells/IL-2 adoptive immunotherapy in combination with chemoradiotherapy for 174 patients with stages I–IV NSCLC [127]. This prospective, randomized, phase III clinical study showed a significant improvement in patient survival among those receiving adjuvant IL-2 LAK immunotherapy. The 5- and 9-year overall survival rates were 54% and 52%, respectively, compared to 33% and 24% in the control group. The subgroup analysis revealed statistically significant differences in the 5-year survival rates, favoring immunotherapy among patients who underwent curative resection. Specifically, the survival rates were 66% for those who received curative resection and 41% for those who did not. Additionally, there were differences in survival rates among patients with different types of cancer: 48% for patients with adenocarcinoma and 23% without, and 62% for patients with squamous-cell carcinoma and 35% without [129,130].

Zhang et al. (2014) assessed the efficacy of adoptive transfer of NK and NKT mixed effector cells in patients with NSCLC [130]. NKT mixed effector cells were generated by expanding PBMCs ex vivo and then phenotypically characterized. The analysis revealed 1.7 times longer overall survival as an outcome measure compared to a control group (31.1 months vs. 18.1 months, *p* = 0.008), indicating a 43.8% reduction in the risk of death. The same significant trend was seen in the 2-year survival rate in the immunotherapy group (62.95% vs. 35.44%, *p* < 0.05). Different independent prognostic factors for patients with NSCLC were revealed in this study. They include clinical stage, gender, the use of tyrosine kinase inhibitors, number of chemotherapy cycles, and, as can be predicted, the application of immunotherapy, which highlights the promising potential of NKT immunotherapy as a treatment option for patients with NSCLC [130,131].

In conclusion, based on a review of the literature, it can be stated that autologous lymphocytes appear to be the most promising for use in adoptive immunotherapy for lung cancer. Hideki Kimura’s preliminary experiments showed that freshly isolated lymphocytes did not display cytotoxicity toward autologous tumor cells over time [131]. However, when cultured in the presence of T-cell growth factor, IL-2, the lymphocytes became cytotoxic toward autologous tumor cells starting on the third day of culture, with peak cytotoxicity occurring on the seventh day [126,127]. A comparative study of the effects of IL-2 and phytohaemagglutinin revealed that while lymphocytes exposed to phytohaemagglutinin exhibited significant cytotoxicity, those exposed to IL-2 demonstrated significantly higher activity [131].

A comprehensive review of clinical trials examining the use of autologous lymphocytes in cancer treatment reveals a consistent trend toward improved overall patient survival among those receiving this approach. In particular, a study conducted by Zhang et al. highlights the significance of both the number of chemotherapy cycles administered and the implementation of EAAL immunotherapy as independent factors contributing to longer survival rates among patients with NSCLC [130]. Through the application of Cox multivariate regression analysis, this study identified a hazard ratio of ~2.8 for the number of chemotherapy cycles (95% confidence interval: 1.157–6.783), indicating that patients undergoing more than six cycles of chemotherapy exhibited significantly higher survival rates compared to those receiving six or fewer cycles. Similarly, a hazard ratio of 3.278 was observed for EAAL immunotherapy (95% CI: 1.415–7.592), indicating a higher likelihood of prolonged survival for patients who received EAAL treatment compared to those in the control group. These findings underscore the promising prospects of autologous lymphocyte infusion immunotherapy in enhancing survival outcomes for patients with NSCLC. The results of the subgroup analysis revealed that the overall survival of patients in the male subgroup with advanced stages receiving chemotherapy for more than six cycles could be significantly prolonged after EAAL cellular immunotherapy. Additionally, the overall survival of other subgroups also showed an improvement after EAAL, although it was not statistically significant [130].

## 7. Discussion

The treatment of lung cancer poses significant challenges due to the complexity of the disease and its heterogeneous nature. In light of the dismal 5-year survival rate for lung cancer of 15–16%, there is an urgent need for innovative treatments. Cancer remains a leading cause of mortality and a major global public health concern, projected to continue as a significant contributor to morbidity and mortality in the decades to come. Bray et al. have estimated that the number of new cancer cases worldwide will rise to 22.2 million by the year 2030 [132]. Recent advances in immunotherapy have highlighted the potential of harnessing the body’s own immune system to combat cancer. Adoptive immunotherapy, whether used as an adjunct or a standalone treatment, holds significant promise for addressing a wide range of malignant tumors [132,133].

A wide variety of cell types, such as NK cells, cytotoxic T lymphocytes, LAK cells, TILs, and activated macrophages, contribute to immune responses that assist in eliminating cancer cells. These cells interact with each other, and their function is modulated by various cytokines. It is, therefore, why the effector cells activated by cytokines are considered an ideal candidate for cancer immunotherapy, including LAK cells, activated NK cells, dendritic cells, activated lymphocytes, TILs, and CIKs—all exhibiting antitumor activity in various contexts. We summarize the main activation factors and anti-tumor mechanisms for these types of cells in Table 1. In addition, immune checkpoints and epigenetic factors contribute to the dynamics and outcomes of balancing between immunosuppression and immune escape of tumors [134].

This review focuses on and extensively discusses the prospects of combined treatment strategies utilizing autologous activated lymphocytes, a promising strategy aimed at enhancing therapeutic efficacy and improving clinical outcomes. Autologous activated lymphocytes are characterized by their ability to elicit robust anti-tumor immune responses. By isolating and subsequently activating a patient’s own lymphocytes, it is possible to augment their capacity to recognize and target neoplastic cells. This personalized approach mitigates the risk of immune rejection while allowing for tailored interventions that are attuned to the unique tumor microenvironment of individual patients. Preliminary clinical studies have reported encouraging findings regarding response rates and overall survival in patients undergoing combined treatment regimens.

Immunotherapy based on activated autologous lymphocytes has advanced significantly in the last 15 years, driven by studies involving various types of interleukins, LAK cells, TILs, and mixed lymphocyte-tumor culture-sensitized cytotoxic T lymphocytes in clinical trials [86,96]. Although the overall tumor shrinkage response rate has been relatively modest at 9%, locoregional administration of TILs into malignant effusions has proven highly effective, achieving 77% shrinkage or elimination of these effusions, even in severely ill patients, resulting in improved quality of life for these individuals [135]. In this review, we summarize the application of autologous lymphocyte therapy in lung cancer treatment, concentrating on the methods of procurement, quality control, and infusion protocols used in >2000 patients. Nevertheless, challenges persist, particularly with regard to the variability in patient responses and the need for optimized methodologies for the activation and expansion of lymphocytes. Additionally, the timing and sequencing of these therapies warrant further investigation to maximize therapeutic benefits.

For example, most methods for ex vivo expansion of the cells have proven challenging, and data supporting prolonged survival remain scarce. Analysis of the clinical benefits of autologous lymphocyte treatment has revealed that T-cells, through cell-to-cell interactions and cytokine activity, influence the initiation, progression, and metastasis of lung cancer, enhancing the overall survival rate of patients. Lymphocyte apheresis, while a promising technique, faces several challenges. Patients undergoing apheresis are often in a weakened state, and their T-cells, being mature and differentiated, may exhibit reduced growth rates. This poses a significant hurdle for ex vivo expansion protocols, as these mature cells may not respond well to the expansion process. Additionally, damaged or weakened T-cells contribute to a lower-quality cell population compared to healthy cells. In addition, tumor cells have multiple ways to evade the immune system. The tumor is heterogeneous and variable during its development. The interactions between cancer cells and immune cells not only create an immunosuppressive microenvironment around the tumor but also create a systemic effect, which reduces the effectiveness of immunotherapy [136,137].

Transfusing an adequate number of lymphocytes that can identify and eliminate tumor cells provides a strong foundation for effective adoptive cell therapy [138]. Earlier research conducted by the authors demonstrated that T-cells from tumor-free hosts can enhance antitumor immunity and alter the harmful balance between tumor cells and the host. Recently, cell therapy based on various types of a cancer patient’s own cells has become an important additional treatment option after surgery, chemotherapy, and radiotherapy. However, the high effect of immunotherapy is achieved through its complex use with generally accepted treatment methods [139]. Nonetheless, utilizing multiple immune cell types has encountered considerable challenges, such as low efficiency and issues with cell expansion. One of the advantages of using autologous activated lymphocytes is that it eliminates ethical issues, since the effector cells are obtained by the usual method of collecting blood from the cubital vein or by apheresis technology [140]. In conclusion, while identifying potential research gaps, we ultimately promote the wider adoption of immune cell therapy as a component of combination strategies for the treatment of lung cancer.

## 8. Future Perspectives

The landscape of lung cancer treatment is evolving, particularly with the integration of immunotherapies and personalized medicine. The combined treatment utilizing autologous activated lymphocytes holds significant promise for improving outcomes in lung cancer patients.

Future studies may explore the synergistic effects of combining autologous activated lymphocytes with existing therapies, such as checkpoint inhibitors, targeted therapies, and traditional chemotherapy. By leveraging multiple mechanisms of action, these combination approaches could lead to improved tumor responses and extended survival rates. As more clinical trials are initiated to test various combinations and techniques involving autologous activated lymphocytes, accumulating real-world evidence will provide insights into the practicality and effectiveness of these treatments. Collaborative efforts among research institutions, pharmaceutical companies, and healthcare providers will be essential to validate findings and inform clinical practice.

Advancements in genomic profiling and biomarker identification will facilitate the customization of treatment regimens with personalized treatment protocols. By selecting patients based on specific tumor characteristics and immune profiles, clinicians can optimize the use of activated lymphocytes, potentially leading to more effective and less toxic therapies.

Ongoing innovations in cellular engineering, such as chimeric antigen receptor (CAR) T-cell technologies, may enhance the effectiveness of autologous lymphocyte treatments. By genetically modifying T-cells for better recognizing and attacking lung cancer cells, researchers could significantly improve patient outcomes. To date, seven CAR T-cell therapies are approved by the U.S. Food and Drug Administration, all designed to treat some blood cancers: six were approved between 2017 and 2022 [141], and the last one targeting B-lymphocyte antigen CD19, Aucatzyl, was approved in November 2024. However, for solid tumors, CAR T-cell therapies are still being designed and tested [142]. Also, despite promising results in treating cancers, challenges persist in the cytotoxicity-related side effects of the CAR T-cell therapies [143]. Recently, alternative or complementary variants of CAR T-cell therapy were proposed and commenced for preclinical studies in solid tumor treatment: CAR-Natural Killer and CAR-macrophage [144]. The preliminary results indicate that CAR-Natural Killer cells favorably substitute for CAR-T-cells because they have lower toxicity and can be obtained from various tissues, including commercially available lines, in contrast to T-cells, which are obtained from the peripheral blood of the patient [145]. On the other hand, the cost for individual CAR T-cell therapies is much higher compared to the therapy based on activated lymphocytes, mostly due to the need to use high-end equipment, techniques, and consumables. The vast majority of the CAR T-cell therapy (pre)clinical studies have been run and remain running in China and the U.S., with fewer such trials in Europe—i.e., in the high-income countries with deeply developed healthcare systems; the average estimate for a single course of such therapy per one patient may be as high as 475,000 USD in the U.S. [146]. Consequently, countries and regions with low- or middle-income economies mostly lack the ability to use this technology. Therefore, an optimal alternative in these circumstances is activated autologous lymphocyte therapy, which provides overall positive results and, in many patients, does slow down or freeze tumor development and progression.

Research into the tumor microenvironment will continue to be crucial. By understanding how cancer cells evade immune detection and how the microenvironment suppresses immune responses, future therapies can be designed to counteract these mechanisms, thereby boosting the efficacy of activated lymphocytes.

As the field progresses, navigating the regulatory landscape will be critical for the approval of new therapies. Moreover, cost-effectiveness analyses will help determine the feasibility of the widespread adoption of these advanced treatments in clinical settings. Future developments will likely emphasize the importance of patient involvement in treatment decision-making. Educational initiatives aimed at informing patients about the benefits and risks of using autologous activated lymphocytes will empower them to participate actively in their care.

Overall, the combined treatment of lung cancer using autologous activated lymphocytes represents a promising frontier in oncology. As research progresses and technology advances, the potential to enhance treatment efficacy, personalize patient care, and improve outcomes will drive the development of this innovative therapeutic approach. Collaborative efforts across disciplines will be essential to realize the full potential of these strategies in the fight against lung cancer.

## Figures and Tables

**Table 1:** Types of activated cells used in immunotherapy and their main in vivo/in vitro factors for their activation as well as anti-tumor mechanisms/factors they exhibit in vivo.

Type of Cells	In Vivo/In Vitro Activation Factors	In Vivo Anti-Tumor Mechanisms and Factors
CD4+ T-cells	TCR + CD28 + anti-CD3/ anti-CD28 antibodies, IFN-γ, TNF-α, TGF-β, IL-4, IL-12, TGF-b, IL-6, IL-23, IL-21	Lyse tumor cells by secretion of IFN-γ, TNF-α Mediate activity of other immune cells and tumor microenvironment by secretion of IFN-γ, TNF-α, GM-CSF, IL3, CXCR3, IL2, IL10 (for Th1 population) IL-4, IL-5, IL-6, IL-9, IL-13, IL-25 (for Th2 population)
CD8+ T-cells	CD3 + CD28, IL-2, IFN-γ, TNF-α, IL-1, IL-12, IL-4, IL-6, IL-21, TGF-β, TRAIL, TCR, CD80/CD86	Lyse tumor cells by perforin, Granzyme B Mediate activity of other immune cells and tumor microenvironment by secretion of IFN-γ, TNF-α, IL-2, IL-12 Cytotoxic via cell-cell interaction Mediate tumor cell apoptosis via Fas/FasL
Natural killer T (NKT) cells	IL-2, IL-15, IL-12, IL-18, TCR, CD1d, lipid stimulation, CD16, NK receptor CD28, CD80/CD86	Lyse tumor cells by perforin, Granzyme B Mediate cytotoxic effects by secretion of IL-2, TNF-α, GM-CSF
Tumor-infiltrating lymphocytes (TILs)	IL-2, CD3, TCR + CD28	Lyse tumor cells by perforin, Granzyme B, IFN-γ, TNF-α Mediate cell-cell interaction and cytotoxic effects by secretion of CD86, CD137-ligand, membrane-bound variant of CD15 Contribute to CXCL-mediated activity
Lymphokine-activated killer (LAK) cells	IL-2, IL-3, IL-4, IL-5, IL-6, IFN-γ, GM-CSF	Lyse tumor cells by perforin, Granzyme B Mediate cytotoxic effects by secretion of IL-2, TNF-α, GM-CSF
Cytokine-induced killer (CIK) cells	IL-2, IL-3, IL-4, IL-5, IL-6, IFN-γ, GM-CSF, CD3, CD16a, interaction via dendritic cells	Lyse tumor cells via TCR/CD3 complex and by cell-cell contact

Abbreviations: NK—natural killer T-cells; TIL—tumor-infiltrating lymphocytes; CIK—cytokine-induced killer; LAK—lymphokine-induced Killer; IL—interleukin; IFN-γ—interferon γ; TNF-α—tumor necrosis factor α; TGF-β—tumor growth factor β; TRAIL—TNF-related apoptosis-inducing ligand; TCR—T-cell receptor; GM-CSF—granulocyte-macrophage colony-stimulating factor; CXCL—chemokine (C-X-C motif) ligand family. Fas—Fas receptor, apoptosis antigen 1; FasL—Fas ligand. Note: neutralizing antibodies are necessary components for in vitro lymphocyte activation.

## Data Availability

Not applicable.

## Abbreviations

CAR	chimeric antigen receptor
CIK	cytokine-induced killer
EAAL	expanded activated autologous lymphocyte
IFN-γ	interferon γ
LAK	lymphokine-activated killer
MHC	major histocompatibility complex
NKT	natural killer T-cells
NSCLC	non-small cell lung cancer
PBMC	peripheral blood mononuclear cells
SCLC	small cell lung cancer
TIL	tumor-infiltrating lymphocyte
